# Nutrient Allocation and Growth Responses of *Senegalia polyphylla* under Varied Fertilizer Regimes for Effective Forest Recovery

**DOI:** 10.3390/plants13172420

**Published:** 2024-08-30

**Authors:** Fillipe Vieira de Araújo, Tayna Sousa Duque, Evander Alves Ferreira, Israel Marinho Pereira, Iasmim Marcella Souza, Fernanda Santos Oliveira, José Barbosa dos Santos

**Affiliations:** 1 Department of Forestry Engineering, Federal University of Vales do Jequitinhonha and Mucuri, Diamantina 39100-000, MG, Brazil; fillipe.vieira10@gmail.com (F.V.d.A.); israel@ufvjm.edu.br (I.M.P.); 2 Department of Agronomy, Federal University of Vales do Jequitinhonha and Mucuri, Diamantina 39100-000, MG, Brazil; evanderalves@gmail.com (E.A.F.); iasmim.marcella@ufvjm.edu.br (I.M.S.); oliveira.fernanda@ufvjm.edu.br (F.S.O.); jbarbosa@ufvjm.edu.br (J.B.d.S.)

**Keywords:** fertilization, resource allocation, exotic grass, biological invasion, nutrients, forest restoration

## Abstract

To restore invaded areas, planting fast-growing native species such as *Senegalia polyphylla* (DC.) Britton & Rose (Fabaceae) is widely used. However, invasive grasses reduce light availability, alter fire regimes, and compete for water and nutrients, hindering the growth of native trees. Fertilization practices influence the competition dynamics between natives and invasives by altering soil fertility. Therefore, this study investigated the effects of mineral and organic fertilization on the nutritional status and growth of *S. polyphylla* cultivated during the first 120 days after transplanting. The experiment was conducted in a completely randomized design comprising five treatments and four replications, along with the unfertilized control (0–0%) as an additional treatment. Dystrophic red latosol and different proportions of mineral and organic fertilizers were used. The variables evaluated included dry mass of aboveground parts and roots, nutrient content in leaves, and nutrient use efficiency. The results showed that fertilizations with high nutrient concentrations (100–0% and 75–25%) resulted in greater accumulation of N, P, and K in the leaves, while balanced fertilization (50–50% and 25–75%) led to greater root dry mass. These results emphasize the importance of strategically choosing fertilizer formulations to promote the healthy development of seedlings in areas subject to interference from invasive grasses.

## 1. Introduction

Biological invasions of plants are detrimental to agricultural and natural environments, being the second leading cause of biodiversity loss worldwide [1]. In Brazil, the Poaceae family is the most abundant group of invasive species [2], and due to their adaptation to climatic conditions and high competitiveness, they are harmful invaders in natural ecosystems [3,4]. These plants can reduce light availability, alter fire regimes, and compete for water and nutrients, limiting the growth of native trees [5,6,7,8].

In the context of restoring invaded areas, planting rapidly growing native species such as *Senegalia polyphylla* (DC.) Britton & Rose (Fabaceae) is widely employed [9,10,11,12]. However, grass management is primarily conducted through chemical control [13,14], which can lead to resurgence before the target species are established [15]. In these systems, fertilization practices can influence the competition dynamics between natives and invaders by altering soil fertility [16,17].

Mineral, organic, and combined fertilizations have been applied in forest restoration, following commercial forestry practices [18]. Few studies have focused on the benefits of combining fertilizers for tropical forest restoration [19] and their impacts related to competition with invasive grasses [17]. Mineral fertilizers applied to the soil are immediately available to plants, and due to their high solubility in water, losses through leaching can favor invasive species over natives [16,20,21]. Organic fertilization is of slow release, being more effective in competitive contexts [22], maintaining nutrient availability at adequate levels for an extended period [23].

Given the high potential of *S. polyphylla* to restore invaded ecosystems, it is necessary to investigate which fertilization practices favor its growth and survival and how they may influence competition with invasive grasses. The hypotheses of this study are as follows: the gradual release of organic nutrients may benefit the growth of *S. polyphylla*, which rapidly creates a canopy and reduces competition with weeds; and the combination of mineral and organic fertilizers may offer an ideal balance between immediate and sustained nutrient availability for the species.

Thus, the aim of this study is to evaluate the effects of mineral and organic fertilization on the nutritional status and growth of *Senegalia polyphylla* during the first 120 days after seedling transplantation.

## 2. Materials and Methods

### 2.1. Experimental Design and Plant Cultivation

The experiment was conducted in a greenhouse located in Diamantina, Minas Gerais, Brazil, at coordinates 18°12′ S and 43°34′ W, at an altitude of 1370 m. The cultivation utilized a substrate of dystrophic red latosol, as described by Santos [24], with medium texture, collected from the surface layer (0–0.20 m). The soil was broken down, air-dried, and sieved through a 5 mm mesh (see Table 1 for details). For chemical and physical analyses, a subsample of 15 cm^3^ was collected and passed through a 2 mm sieve, following the method of Teixeira [25].

Seedlings of *Senegalia polyphylla* originated from seeds collected in the Private Natural Heritage Reserve of Fartura, Minas Gerais, Atlantic Forest Biome (15°30′ S; 39°50′ W). After processing, the seeds were planted in tubes containing 0.29 dm^3^ of a substrate composed of sterilized soil, carbonized rice husk, and sand (1:1:1). Seedlings with four pairs of leaves and an average height of 0.08 m were selected. After removal from the tubes, the seedlings had their roots washed and were transplanted into polyethylene pots containing 6 dm^3^ of soil.

The experimental design was completely randomized, with five main treatments and one additional treatment, each with four replications. The treatments consisted of different proportions of mineral (MF) and organic (OF) fertilizers for *S. polyphylla* (Table 2). The additional treatment represented the condition of 0% for both forms of fertilization (0–0%).

Mineral fertilization (100–0%) was applied with 50 mg of N (ammonium sulfate), 150 mg of P (single superphosphate), and 50 mg of K (potassium chloride) per dm^3^ of soil [26]. Organic fertilization (0–100%) used 5 g of cured cattle manure per dm^3^ of soil [27]. The nutrients in the organic compost were analyzed using nitroperchloric digestion [28], showing levels of 3.1% N, 1.8% P, 2.1% K, 12.3% Ca, 2.1% Mg, and 26.1% S. The other treatments received different proportions of mineral and organic fertilizations, applied 15 days before transplanting the seedlings or planting the grass.

Deionized water was added daily to maintain soil moisture around 60% of the total pore volume from the fertilization period to the end of the experiment.

### 2.2. Measured Variables

In the evaluation of variables, aboveground and root parts were collected, washed, and treated according to Silva [28]. The collected plant material was dried in a forced air circulation oven at 65 °C until constant mass, allowing the determination of the dry mass of leaves (DML), stem (DMST), total aboveground part (MSPA), and roots (DMR).

The nutrient content in the leaves was determined using sulfuric and nitroperchloric digestion methods, as per Silva [28]. The total nutrient accumulation in the leaves was calculated based on the nutrient levels and the dry mass of the leaves.

The efficient use (EU) of nutrients for the production of aboveground mass (MSPA) was obtained from the ratio between MSPA and the accumulation of N, P, K, Ca, Mg, and S. To determine sufficiency ranges, leaf tissue analyses were compared with nutritional sufficiency intervals of other species, given the absence of specific values for *S. polyphylla* (Table 3). A literature review included studies on fast-growing trees in similar substrates [29].

To analyze the response of *S. polyphylla* to different fertilizations, vector analysis was employed to assess the dry mass and nutritional status of the leaves, expressed in relation to the control group (0–0%), normalized to 100.

### 2.3. Statistical Analysis

Statistical analysis used the “ExpDes.pt” package in the R version 4.3.2 environment [33]. Before ANOVA and regression analyses, data were tested for normality of distribution using the Shapiro–Wilk test on residuals. All data were confirmed to be normally distributed. An α = 0.05 was used to determine statistical significance in all analyses.

To evaluate the impact of the gradual reduction in mineral fertilization and increase in organic fertilization on the availability and use of nutrients, the leaf content (%) and efficient use data were subjected to polynomial regression.

To determine if the gradual change impacted the production of dry mass and to identify which fertilization groups exhibited different results, the Scott–Knott test was utilized for DML, DMST, DMR, DMSH, and DMT. The Dunnett test was conducted to determine whether the dry mass from a specific fertilization (F) was significantly higher than that of no fertilization (0–0%) (C).

## 3. Results

### 3.1. Nutrient Use Efficiency Results

**Figure 1:**
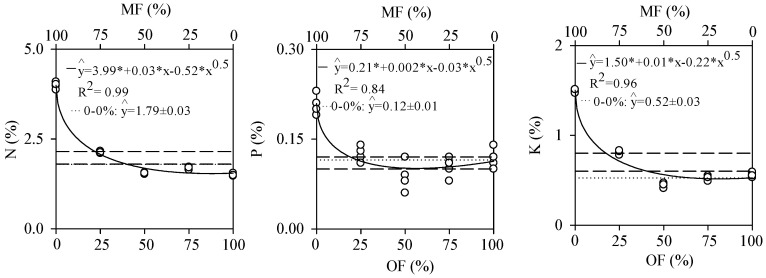
Leaf contents (%) of nitrogen (N), phosphorus (P), potassium (K) of *Senegalia polyphylla* in non-competitive cultivation 120 days after transplanting in a substrate containing dystrophic red latosol. The solid line (-) represents leaf content values in mineral (MF) and organic (OF) fertilizations, the dotted (...) the control (0–0%), and the dashed (--) the upper and lower limits of sufficiency ranges. The upper and lower axes, per graph, show the combinations of MF and OF. * significant at 5% via the F test.

The analysis of the responses of *Senegalia polyphylla* to different fertilization regimes revealed that the foliar content of nitrogen (N), phosphorus (P), and potassium (K) was significantly higher with 100% mineral fertilization and 0% organic fertilization (Figure 1).

**Figure 2:**
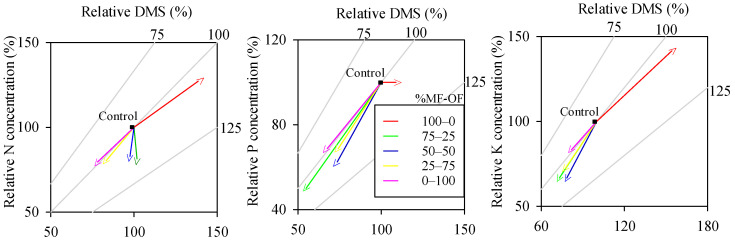
Vector diagrams of changes in the dry mass of the aboveground part (DMS), nitrogen (N), phosphorus (P), and potassium (K) content and accumulation in relation to the unfertilized treatment (0–0%) of *Senegalia polyphylla* in non-competitive cultivation 120 days after transplanting in a substrate containing dystrophic red latosol. Values of the control treatment were normalized to 100%.

Treatments with 100% and 75% mineral fertilization resulted in higher concentrations of N, P, and K and consequently greater biomass accumulation in the leaves of *S. polyphylla*. Treatments with 50%, 25%, and 0% mineral fertilization caused reductions in nutrient accumulation compared to the control treatment (0–0%) (Figure 2).

**Figure 3:**
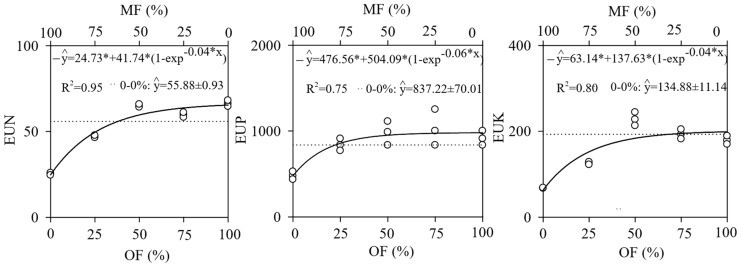
Efficiency of the use (EU) of nitrogen (EUN), phosphorus (EUP), and potassium (EUK) by *Senegalia polyphylla* in non-competitive cultivation at 120 days after transplanting in a substrate containing dystrophic red latosol. The solid line (-) represents EU values in mineral (MF) and organic (OF) fertilizations, and the dotted (...), the control (0–0%). The upper and lower axes, per graph, show the combinations of MF and OF. * significant at 5% via the F test.

The use efficiency (EU) of N, P and K to produce dry mass of leaves was higher in treatments with higher organic fertilization (0–100%) (Figure 3).

### 3.2. Growth Characteristics Results

**Figure 4:**
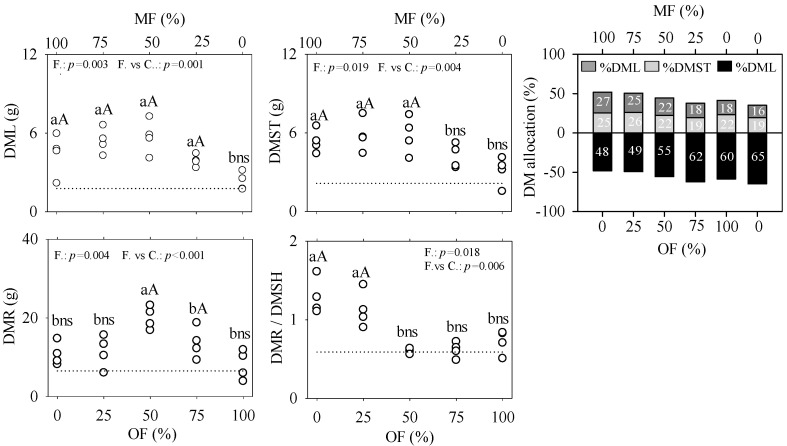
Dry mass of leaves (DML), stem (DMST), root (DMR), aboveground/belowground ratio DMSH/DMR, and percentage of total dry mass allocation of *Senegalia polyphylla* in non-competitive cultivation at 120 days after transplanting in a substrate containing dystrophic red latosol. The dashed line (...) represents the dry mass values of the control (0–0%). The upper and lower axes, per graph, show the combinations of MF and OF. Lowercase letters differ between fertilizations (F) at 5% via the Scott–Knott test. Uppercase letters differ between fertilizations (F) in relation to the control (C) (0–0%) at 5% using the Dunnett test.

The dry leaf mass (DML) was lower in treatments with 100% organic fertilizer. In treatments with 50% and 75% organic fertilizer, the reduction in dry leaf mass was less pronounced, and the dry root mass (DMR) was higher, leading to a lower aboveground/root ratio (DMSH/DMR) (Figure 4).

**Figure 5:**
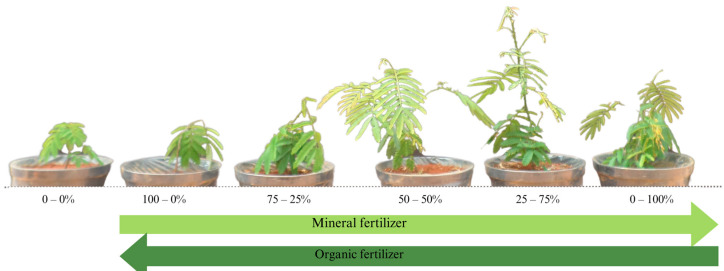
*Senegalia polyphylla* plant cultivation 120 days after transplanting in substrate containing dystrophic red latosol with different proportions of mineral and organic fertilizers.

Images of *Senegalia polyphylla* were acquired 120 days after transplantation in a substrate composed of dystrophic red latosol with varying proportions of mineral and organic fertilizers (Figure 5).

## 4. Discussion

### 4.1. Nutrient Use Efficiency

The foliar content of N, P, and K was higher with mineral fertilization (100–0%). This occurred because mineral fertilizers are generally composed of water-soluble salts that provide nutrients immediately and in high concentrations, without relying on decomposition or mineralization processes [20]. This rapid availability results in an elevated absorption of N, P, and K, promoting a quick increase in foliar nutrient levels. However, elevated foliar nutrient levels with mineral fertilization are not always related to efficient resource use, potentially resulting in losses through leaching [21,34]. Additionally, the immediate provision of nutrients leads to reduced investment in the development of extensive root systems, as nutrients are readily accessible [35].

Treatments subjected to 50%, 25%, and 0% mineral fertilization had reductions in N, P, and K in the leaves compared to the control. This can be attributed to the lower immediate availability of these nutrients in the soil. With the reduction in the proportion of mineral fertilizers, the amount of readily soluble and available nutrients for plant absorption is also reduced [22,23]. The concentration of nutrients in the leaves is essential for allowing plants to accumulate reserves during periods of high availability and gradually release them during scarcity to sustain growth [36]. Therefore, the excessive use of fertilizers is common to increase the survival of native tree seedlings and reduce competition from invasive grasses in forest restoration [16,21].

The efficiency of the use of N, P, and K was higher in treatments with organic fertilization. This occurred because, generally with nutrient scarcity, greater efficiency with their use is observed [34,37]. Organic fertilization, compared to mineral fertilization, allows a slow release of nutrients; thus, N, P, and K are not immediately available to *S. polyphylla* [38]. These characteristics favor root growth in the species, aiming for greater soil exploration, absorption, and nutrient use [35,39]. On the other hand, mineral fertilization provides nutrients immediately, which may not be fully utilized by the plant, thus reducing the efficiency of the use of N, P, and K in these treatments [40].

### 4.2. Growth Characteristics

The lower dry leaf mass in the 100% organic fertilization treatment and the increased root dry mass in the treatments with 50% and 75% organic fertilizer can be explained by the functional balance hypothesis. This hypothesis suggests a preferential allocation of resources to the root system when nutrients become limiting for growth [41], correlating with the slow nutrient release in organic fertilization. Allocating resources to root development can facilitate the exploration of deeper soil layers, providing a competitive advantage to tree seedlings during the initial stages of development [42,43].

Given the presence of invasive grasses, fertilization plays a crucial role in determining the initial success of forest restoration and the establishment of seedlings in the field [44]. The rapid establishment of a canopy is a primary goal in tropical forest restoration, as reducing light availability in the understory inhibits the growth of shade-intolerant invasive grasses [36]. Therefore, the strategic application of fertilizers can boost the initial growth of trees [45]; however, it is necessary to consider the variations in the individual species’ responses [9,46].

Considering the persistence of invasive grasses and the lack of internal nutrient reserves in *S. polyphylla*, additional nutrient supplementation, especially in the early stages of planting, may be a viable strategy to sustain growth and minimize competition from invasive plants. The slow-release nature of organic fertilizers can ensure a steady supply of nutrients, promoting a more balanced growth that includes extensive root development, which is crucial for long-term sustainability and a competitive advantage in invaded ecosystems.

## 5. Conclusions

This study evaluated the effects of mineral and organic fertilization on the nutritional status and growth of *S. polyphylla*, highlighting that mineral fertilization resulted in greater foliar nutrient accumulation and biomass. In contrast, organic fertilization promoted higher nutrient use efficiency, favoring the development of the root system and enabling more efficient soil exploration. These findings indicate that the strategic combination of fertilizers can optimize the initial growth of *S. polyphylla*, reducing competition with invasive grasses and promoting more sustainable forest restoration.

Thus, the application of fertilizers should be carefully planned to maximize nutrient use efficiency and ensure balanced plant growth, focusing both on rapid canopy formation and root development. The use of organic fertilizers can ensure a continuous supply of nutrients, essential for long-term sustainability and a competitive advantage in invaded ecosystems.

## Figures and Tables

**Table 1:** Chemical and physical soil analysis before the application of organic and mineral fertilization treatments.

pH Water	P	K	Ca	Mg	Al	T	m	V	CO	Sand	Silt	Clay
1:2.5	-mg dm^−3^-		----cmol_c_ dm^−3^----				--%--		------------g kg^−1^-----------			
6.0	0.4	15.9	2.5	0.4	0.02	7.1	1	41	1.0	460	230	310

pH water: Soil–water ratio 1:2.5. P and K: Mehlich 1 extractor. Ca, Mg, and Al: KCl extractor 1 mol L-1. T: Cation exchange capacity at pH 7.0. m: Aluminum saturation. V: Base saturation. CO: Organic carbon using the Walkey–Black method. Sand, silt, and clay: Pipette method.

**Table 2:** Amounts of nitrogen (N), phosphorus (P), potassium (K), calcium (Ca), magnesium (Mg), sulfur (S), and organic carbon (OC) applied from mineral (MF) and organic (OF) fertilizers for *Senegalia polyphylla* in typical dystrophic red latosol.

MF–OF	N	P	K	Ca	Mg	S	CO
--%--	-------------------------mg dm^−3^-------------------------					-----g dm^−3^-----	
0–0	0–0	0–0	0–0	0–0	0–0	0–0	0–0
100–0	50–0	150–0	50–0	383–0	50–0	0.3–0	0–0
75–25	38–39	113–10	38–15	330–110	45–15	0.5–0.2	0.0–0.7
50–50	25–78	75–20	25–35	206–206	35–35	0.4–0.4	0–1.4
25–75	13–117	38–30	13–58	105–317	19–58	0.3–0.8	0–2.1
0–100	0–155	0–40	0–87	0–437	0–87	0–1.3	0–2.7

**Table 3:** Sufficiency ranges found in the literature for macronutrients in the leaves of *Senegalia polyphylla*.

N	P	K	Ca	Mg	S
------------------------Sufficiency Ranges (%)------------------------					
2.00–3.00	0.10–0.12	1.00–1.20	0.40–0.50	0.15–0.30	0.15–0.20

References: *S. polyphylla*: N—[30]; P—[31]; K—[32].

## Data Availability

Data are contained within the article.

## References

[1] Adelino J.R.P., Heringer G., Diagne C., Courchamp F., Faria L.D.B., Zenni R.D. (2021). The economic costs of biological invasions in Brazil: A first assessment. NeoBiota.

[2] Zenni R.D., Ziller S.R., da Rosa C.A., Sühs R.B., Puechagut P.B., Marterer B.T., Chapla T.E. (2024). Invasive non-native species in Brazil: An updated overview. Biol. Invasions.

[3] Canavan S., Meyerson L.A., Packer J.G., Pyšek P., Maurel N., Lozano V., Richardson D.M., Brundu G., Canavan K., Cicatelli A. (2019). Tall-statured grasses: A useful functional group for invasion science. Biol. Invasions.

[4] Gornish E.S., Franklin K., Rowe J., Barberán A. (2020). Buffelgrass invasion and glyphosate effects on desert soil microbiome communities. Biol. Invasions.

[5] Assis G.B., Pilon N.A., Siqueira M.F., Durigan G. (2021). Effectiveness and costs of invasive species control using different techniques to restore cerrado grasslands. Restor. Ecol..

[6] Dezotti G., Fidelis A., Damasceno G., Siqueira T. (2024). Interactive roles of fire seasons and biological invasions in the short-term dynamics of tropical savannas. J. Veg. Sci..

[7] Damasceno G., Souza L., Pivello V.R., Gorgone-Barbosa E., Giroldo P.Z., Fidelis A. (2018). Impact of invasive grasses on Cerrado under natural regeneration. Biol. Invasions.

[8] Mazía N., Moyano J., Perez L., Aguiar S., Garibaldi L.A., Schlichter T. (2016). The sign and magnitude of tree–grass interaction along a global environmental gradient. Glob. Ecol. Biogeogr..

[9] Almeida C., Viani R.A. (2019). Selection of shade trees in forest restoration plantings should not be based on crown tree architecture alone. Restor. Ecol..

[10] Meli P., Isernhagen I., Brancalion P.H., Isernhagen E.C., Behling M., Rodrigues R.R. (2018). Optimizing seeding density of fast-growing native trees for restoring the Brazilian Atlantic Forest. Restor. Ecol..

[11] Souza M.T.P., Azevedo G.B., Azevedo G.T.D.O.S., Teodoro L.P.R., Plaster O.B., Assunção P.C.G., Teodoro P.E. (2020). Growth of native forest species in a mixed stand in the Brazilian Savanna. For. Ecol. Manag..

[12] Barbosa R.S., Vale R.S., Schwartz G., Martins W.B.R., Ribeiro S.S., Rodrigues J.I.M., Ferreira G.C., Barbosa V.M. (2022). Restoration of degraded areas after bauxite mining in the eastern Amazon: Which method to apply?. Ecol. Eng..

[13] Miller-Adamany A., Baumann D., Thomsen M. (2019). Facilitating natural succession in a heavily invaded ecosystem. For. Ecol. Manag..

[14] Thomas P.A., Schüler J., Boavista L.D.R., Torchelsen F.P., Overbeck G.E., Müller S.C. (2018). Controlling the invader *Urochloa decumbens*: Subsidies for ecological restoration in subtropical Campos grassland. Appl. Veg. Sci..

[15] Massa D., Benvenuti S., Cacini S., Lazzereschi S., Burchi G. (2019). Effect of hydro-compacting organic mulch on weed control and crop performance in the cultivation of three container-grown ornamental shrubs: Old solutions meet new insights. Sci. Hortic..

[16] Pokharel P., Kwak J.H., Chang S.X. (2017). Growth and nitrogen uptake of jack pine seedlings in response to exponential fertilization and weed control in reclaimed soil. Biol. Fertil. Soils.

[17] Krapfl K.J., Hatten J.A., Roberts S.D., Baldwin B.S., Rousseau R.J., Shankle M.W. (2016). Capacity of biochar application and nitrogen fertilization to mitigate grass competition upon tree seedlings during stand regeneration. For. Ecol. Manag..

[18] Brancalion P.H., Campoe O., Mendes J.C.T., Noel C., Moreira G.G., Melis J., Noel C., Stape J.L., Guillemot J. (2019). Intensive silviculture enhances biomass accumulation and tree diversity recovery in tropical forest restoration. Ecol. Appl..

[19] Ornelas A.C.S., Providello A., Soares M.R., Viani R.A.G. (2022). Silvicultural intensification has a limited impact on tree growth in forest restoration plantations in croplands. For. Ecol. Manag..

[20] Tremblay P.Y., Thiffault E., Pinno B.D. (2019). Effects of land reclamation practices on the productivity of young trembling aspen and white spruce on a reclaimed oil sands mining site in northern Alberta. New For..

[21] Schott K.M., Snively A.E., Landhäusser S.M., Pinno B.D. (2016). Nutrient loaded seedlings reduce the need for field fertilization and vegetation management on boreal forest reclamation sites. New Forests.

[22] Otero M., Salcedo I., Txarterina K., González-Murua C., Duñabeitia M.K. (2019). Quality assessment of *Pinus radiata* production under sustainable nursery management based on compost tea. J. Plant Nutr. Soil Sci..

[23] Arif M.S., Shahzad S.M., Riaz M., Yasmeen T., Shahzad T., Akhtar M.J., Bragazza L., Buttler A. (2017). Nitrogen-enriched compost application combined with plant growth-promoting rhizobacteria (PGPR) improves seed quality and nutrient use efficiency of sunflower. J. Plant Nutr. Soil Sci..

[24] Santos H.G., Jacomine P.K.T., Anjos L.H.C., Oliveira V.A., Lumbreras J.F., Coelho M.R., Almeida J.A., Cunha T.J.F., Oliveira J.B. (2018). Sistema Brasileiro de Classificação de Solos.

[25] Teixeira P.C., Donagemma G.K., Fontana A., Teixeira W.G. (2017). Manual de Métodos de Análise de Solo.

[26] Gonçalves E.O., Paiva H.N., Neves J.C.L., Gomes J.M. (2008). Crescimento de mudas de angico-vermelho (*Anadenanthera macrocarpa* (Benth.) Brenan) sob diferentes doses de macronutrientes. Rev. Árvore.

[27] Ribeiro A.C., Guimarães P.T.G., Alvarez V.H., CFSEMG (Comissão de Fertilidade do Solo do Estado de Minas Gerais) (1999). Adubação orgânica. Recomendações para o Uso de Corretivos e Fertilizantes em Minas Gerais.

[28] Silva F.C. (2009). Manual de Análises Químicas de Solos, Plantas e Fertilizantes.

[29] O’Brien P.L., Thomas A.L., Sauer T.J., Brauer D.K. (2020). Foliar nutrient concentrations of three economically important tree species in an alley-cropping system. J. Plant Nutr..

[30] Voigtlaender M., Brandani C.B., Caldeira D.R.M., Tardy F., Bouillet J.P., Gonçalves J.L.M., Moreira M.Z., Leite F.P., Brunet D., Paula R.R. (2019). Nitrogen cycling in monospecific and mixed-species plantations of Acacia mangium and Eucalyptus at 4 sites in Brazil. For. Ecol. Manag..

[31] Manghabati H., Kohlpaintner M., Ettl R., Mellert K., Blum U., Göttlein A. (2018). Correlating phosphorus extracted by simple soil extraction methods with foliar phosphorus concentrations of *Picea abies* (L.) H. Karst. and *Fagus sylvatica* (L.). J. Plant Nutr. Soil Sci..

[32] Lamontagne M., Adégbidi H.G., Assamoi A.J. (2019). Organic fertilization of Christmas tree (*Abies balsamea* (L.) Mill.) plantations with poultry manure in northwestern New Brunswick, Canada. For. Chron..

[33] Ferreira E.B., Cavalcanti P.P., Nogueira D.A. (2014). ExpDes: An R package for ANOVA and experimental designs. Appl. Math..

[34] Achat D.L., Pousse N., Nicolas M., Augusto L. (2018). Nutrient remobilization in tree foliage as affected by soil nutrients and leaf life span. Ecol. Monogr..

[35] De Souza Kulmann M.S., Arruda W.S., Vitto B.B., de Souza R.O.S., Berghetti Á.L.P., Tarouco C.P., Araújo M.M., Nicoloso F.T., Schumacher M.V., Brunetto G. (2021). Morphological and physiological parameters influence the use efficiency of nitrogen and phosphorus by Eucalyptus seedlings. New For..

[36] Zhao Q., Zeng D.H. (2019). Nitrogen addition effects on tree growth and soil properties mediated by soil phosphorus availability and tree species identity. For. Ecol. Manag..

[37] Lima Neto A.J., Neves J.C.L., Martinez H.E.P., Sousa J.S., Fernandes L.V. (2021). Nutrient accumulation and nutritional efficiency in eucalyptus. J. Plant Nutr..

[38] Morais E.G., Silva C.A. (2023). Novel slow-release NPK biochar-based fertilizers with acidulated apatite: Evaluation of the fertilization value in a short-term experiment. J. Soil Sci. Plant Nutr..

[39] De Souza Kulmann M.S., Stefanello L.O., Arruda W.S., Sans G.A., Parcianello C.F., Hindersmann J., Berghetti A.L.P., Araujo M.M., Gatiboni L.C., Brunetto G. (2020). Nitrogen supply methods affect the root growth dynamics in *Eucalyptus grandis*. For. Ecol. Manag..

[40] Chatzistathis T., Kavvadias V., Sotiropoulos T., Papadakis I.E. (2021). Organic fertilization and tree orchards. Agriculture.

[41] Jaquetti R.K., Nascimento H.E.M., Zotarelli L., Rathinasabapathi B., Carvalho J.F.G. (2022). Coordinated adjustments of carbohydrates and growth of tree legumes under different fertilization regimes in degraded areas in Amazonia. New For..

[42] Andivia E., Villar-Salvador P., Oliet J.A., Puertolas J., Dumroese R.K., Ivetić V., Molina-Venegas R., Arellano E.C., Ovalle J.F. (2021). Climate and species stress resistance modulate the higher survival of large seedlings in forest restorations worldwide. Ecol. Appl..

[43] Pillay T., Ngcobo S., Ward D. (2023). Nutrient addition, fire and grass competition affects biological nitrogen fixation in Vachellia sieberiana, and associated soil respiration. Pedobiologia.

[44] Shi Z., Bai Z., Guo D., Li S., Chen M. (2023). Species Diversity and Soil Interconstraints Exert Significant Influences on Plant Survival during Ecological Restoration in Semi-Arid Mining Areas. Diversity.

[45] Sloan J.L., Uscola M., Jacobs D.F. (2016). Nitrogen recovery in planted seedlings, competing vegetation, and soil in response to fertilization on a boreal mine reclamation site. For. Ecol. Manag..

[46] Maciel J.C., Duque T.S., Ferreira E.A., Zanuncio J.C., Plata-Rueda A., Silva V.P., dos Santos J.B. (2022). Growth, Nutrient Accumulation, and Nutritional Efficiency of a Clonal Eucalyptus Hybrid in Competition with Grasses. Forests.

